# From Clinical Suspicion to Therapeutic Resolution: A Case of Melkersson-Rosenthal Syndrome

**DOI:** 10.7759/cureus.90763

**Published:** 2025-08-22

**Authors:** Omar Gerardo Galaviz-Chaparro, José Alfredo Soto-Ortiz, Claudia Velázquez-Rodríguez, Andrea Sierra-Franco, María Fernanda Limón-Limón

**Affiliations:** 1 Dermatology, Instituto Dermatológico de Jalisco "Dr. José Barba Rubio", Zapopan, MEX; 2 Dermatology, University of Guadalajara, Zapopan, MEX

**Keywords:** granulomatous cheilitis, intralesional steroids, lip swelling, melkersson-rosenthal syndrome, orofacial granulomatosis

## Abstract

Melkersson-Rosenthal syndrome (MRS) is a rare, chronic condition characterized by orofacial edema, facial paralysis, and fissured tongue. Its etiology remains unclear, although genetic, immunological, and infectious factors have been proposed in its pathogenesis. Various treatment modalities have been suggested, including corticosteroids, antibiotics, immunosuppressants, and, in severe cases, surgical intervention. Early identification is essential to improve prognosis and prevent complications. We report the case of a 33-year-old woman presenting with progressive and persistent oral edema accompanied by a fissured tongue. Histopathological examination confirmed granulomatous cheilitis, leading to the diagnosis of MRS. Treatment with intralesional triamcinolone acetonide and doxycycline was initiated, resulting in an excellent clinical response.

## Introduction

Melkersson-Rosenthal syndrome (MRS), first described in 1928 by Ernst Gustaf Melkersson, is a rare granulomatous disorder characterized by the classic triad of orofacial edema, facial paralysis, and fissured tongue [1]. Symptom onset typically occurs between the ages of 25 and 40, with female predominance, while pediatric cases are uncommon and usually appear between seven and 12 years of age. Diagnosis is frequently delayed, with a mean interval of four to nine years before confirmation. Management is multidisciplinary and may include corticosteroids, antibiotics, and, in severe or refractory cases, immunosuppressants or surgical intervention [2-4]. Early recognition is crucial to improve outcomes and prevent complications. This case is particularly relevant as it highlights the diagnostic challenges of MRS, emphasizes the importance of clinicopathological correlation, and demonstrates a favorable therapeutic response, thereby contributing to the limited literature on effective diagnostic and management strategies for this rare condition.

## Case presentation

A 33-year-old woman presented for medical evaluation with a one-year history of progressive and persistent edematous cheilitis, which caused difficulty in speech articulation, as well as a fissured tongue. No neurological abnormalities were observed. Her medical history included systemic arterial hypertension and type 2 diabetes mellitus, both under treatment and control.

**Figure 1:**
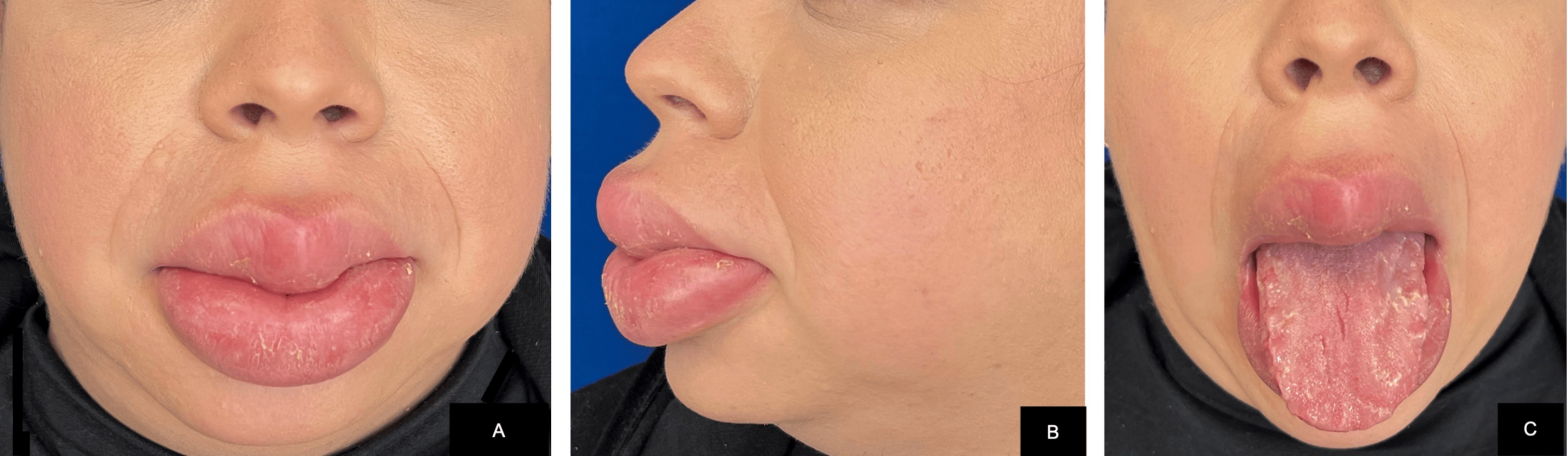
Swelling with edema and induration of both lips (A and B); fissured tongue (C).

Physical examination revealed involvement of the oral cavity, with swelling and induration of both the upper and lower lips. The dorsal surface of the tongue showed numerous irregular fissures following the anatomical contours, along with detachable whitish plaques. The remainder of the physical examination was unremarkable (Figure 1).

**Figure 2:**
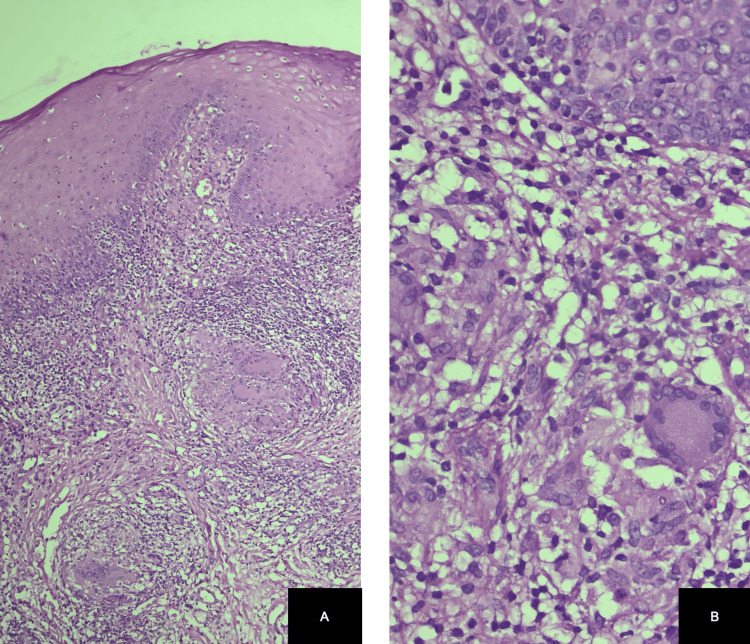
Granulomatous cheilitis showing acanthotic epithelium, dilated vessels in the chorion, and non-caseating granulomas composed of histiocytes, multinucleated giant cells, and lymphocytic infiltrate.

An incisional biopsy was performed using a 4 mm punch on the upper lip mucosa, obtaining epithelium and underlying connective tissue for histopathological evaluation. Histopathological examination revealed acanthotic epithelium, dilated vessels in the chorion, and non-caseating granulomas composed of histiocytes, multinucleated giant cells, and a lymphocytic infiltrate, findings consistent with granulomatous cheilitis (Figure 2). The clinicopathological correlation led to the diagnosis of MRS.

**Figure 3:**
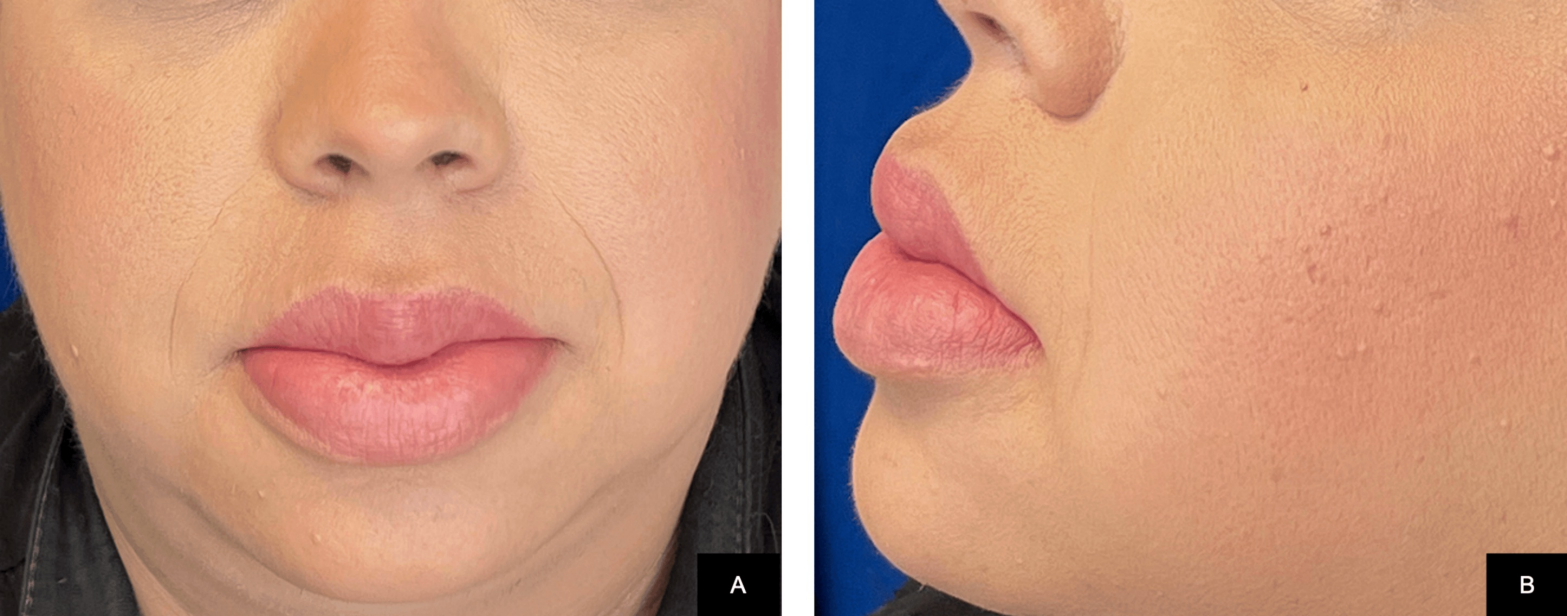
Reduction of lip edema three months after initiation of therapy.

Treatment was initiated with intralesional triamcinolone acetonide, 40 mg weekly, until reaching a cumulative dose of 120 mg per lip, along with doxycycline 100 mg/day. The patient showed a favorable clinical response at three months after initiation of therapy, evidenced by a reduction in lip volume and improvement in speech articulation (Figure 3).

## Discussion

The etiology of MRS remains unclear. Proposed mechanisms include neural abnormalities, chronic infections such as herpes simplex, and hypersensitivity reactions to bacterial antigens. Associations with systemic conditions, including Crohn’s disease and sarcoidosis, have also been reported. A genetic predisposition has been suggested, with variants in the ATP1 gene, involved in fatty acid transport, and a heterozygous mutation in the SLC27A1 (FATP1) gene identified as potential contributors [2].

Clinically, MRS follows a chronic course with periods of remission and relapse, and in some cases, spontaneous resolution [2]. Age of onset ranges from childhood to late adulthood, with a female predominance (2:1) [3,4]. The syndrome is classically defined by the triad of facial paralysis, ipsilateral facial edema, and fissured tongue, although the latter occurs in only 25-30% of cases [2].

Facial edema is the most common manifestation, observed in 86% of cases [2]. In a retrospective study by Elias et al., facial swelling was present in all patients, with isolated lip involvement in 74% and the complete triad in only 13% of cases [5]. The clinical presentation typically begins with edema of one or both lips, eyelids, and, less commonly, one side of the scalp. The upper lip is the most frequently affected site [6]. When the inflammation is restricted to the lips, the condition is referred to as Miescher’s cheilitis [2]. Orofacial inflammation is often the initial finding and may be confused with hereditary or acquired angioedema, a disorder characterized by histamine- or bradykinin-mediated plasma leakage affecting subcutaneous and/or submucosal tissues [3]. In our patient, bilateral lip edema and a fissured tongue were evident; the latter is defined by grooves on the dorsal surface of the tongue measuring at least 2 mm in depth and 15 mm in length [4].

Facial paralysis may precede or follow the edema, with an interval ranging from months to years. It is observed in 47-90% of cases and may be transient or, less commonly, permanent. It is typically unilateral, though bilateral involvement is rare; the recurrence rate is approximately 10% [7].

Diagnosis is often delayed by four to nine years [2] and is primarily clinical, based on the presence of the classic triad [4]. A diagnosis can be established when at least two of these features are present. When only two manifestations are observed, the condition is classified as oligosymptomatic MRS; if only one is present, it is considered monosymptomatic. In such cases, diagnosis is confirmed by biopsy, which reveals non-necrotizing granulomatous cheilitis in patients with isolated facial or lip edema, also known as Miescher’s variant of MRS [8].

Histopathologically, lip biopsies may reveal a lymphomononuclear infiltrate, non-caseating granulomas composed of epithelioid cells, multinucleated Langerhans-type giant cells, and fibrosis [3]. In early disease stages, the infiltrate consists predominantly of lymphocytes and plasma cells, which evolve into granulomatous infiltrates and eventually fibrosis [9]. Given the frequent diagnostic delay, histopathological analysis of a lip biopsy is essential for confirmation [3].

The differential diagnosis of MRS includes chronic inflammatory and infectious diseases that present with granulomatous infiltrates, as well as rosacea, contact dermatitis, allergic reactions, foreign body responses, and Bell’s palsy [3].

Management of MRS is challenging and often requires a multidisciplinary approach. Treatment may involve corticosteroids, antibiotics, and antidepressants [6]. Intralesional triamcinolone is effective for orofacial edema, typically administered at doses of 10-40 mg/mL with weekly injections titrated to clinical response, and booster doses after six months if needed. Our patient responded after three weekly doses. Doxycycline and minocycline may reduce inflammation, and their combination with corticosteroids enhances the therapeutic effect. Oral corticosteroids are recommended in cases with facial paralysis, while intralesional injections are preferred for isolated or refractory edema [4].

For patients who do not respond to initial therapy, immunosuppressive agents may be considered [2]. In systemic cases, methotrexate, thalidomide, intravenous immunoglobulin, and anti-TNF agents such as infliximab and adalimumab have been used [4].

In severe or refractory cases, surgical options have been described [2]. For recurrent facial paralysis, facial nerve decompression may reduce the risk of relapse. In cases of persistent lip edema, cheiloplasty or laser ablation may be performed, although these procedures may impair sensitivity and do not prevent recurrence [4].

This case highlights the diagnostic challenges of MRS, underlining the importance of biopsy and clinicopathological correlation in establishing the diagnosis. Moreover, the favorable therapeutic response observed at three months underscores the effectiveness of combined intralesional corticosteroid and antibiotic therapy in achieving functional and aesthetic improvement. These findings contribute valuable clinical insights to the limited literature on MRS and may assist clinicians in improving recognition, timely diagnosis, and therapeutic decision-making in this rare condition.

## Conclusions

MRS is a rare neuromucocutaneous disorder that requires timely diagnosis to initiate appropriate treatment and prevent recurrences and complications. Histopathological confirmation of granulomatous cheilitis is essential for establishing the diagnosis. A variety of therapeutic options are available, and their selection should be guided by the clinical presentation and disease severity. Favorable outcomes are more likely when early diagnosis and prompt intervention are achieved. In the present case, the patient exhibited progressive resolution of lip edema and marked improvement in speech articulation following treatment with intralesional triamcinolone acetonide and oral doxycycline. No recurrence was documented during follow-up. Ongoing monitoring is advised due to the potential risk of relapse.
